# The Anti-Inflammatory Mechanism of Flaxseed Linusorbs on Lipopolysaccharide-Induced RAW 264.7 Macrophages by Modulating TLR4/NF-κB/MAPK Pathway

**DOI:** 10.3390/foods12122398

**Published:** 2023-06-16

**Authors:** Jialong Li, Jing Chen, Ping Huang, Zizhe Cai, Ning Zhang, Yong Wang, Ying Li

**Affiliations:** 1 Guangdong International Joint Research Center for Oilseeds Biorefinery, Nutrition and Safety, Department of Food Science and Engineering, Jinan University, Guangzhou 510632, China; jialongli@stu2021.jnu.edu.cn (J.L.); chenj@jnu.edu.cn (J.C.); caizz@jnu.edu.cn (Z.C.); tzhning@jnu.edu.cn (N.Z.); twyong@jnu.edu.cn (Y.W.); 2 Institute for Advance and Application Chemical Synthesis, Jinan University, Guangzhou 510632, China; 3 Guangzhou Meizhiao Cosmetics Co., Ltd., No. 555, Panyu Av. North, Guangzhou 510000, China; huang.ping@meizhiao.com

**Keywords:** flaxseed linusorbs, anti-inflammatory mechanism, TLR4/NF-κB/MAPK pathway, raw 264.7 macrophage, inflammatory cytokines

## Abstract

Flaxseed linusorbs (FLs), cyclic peptides derived from flaxseed oils, have shown multiple activities such as anticancer, antibacterial, and anti-inflammatory effects. However, the anti-inflammatory monomers of FLs and their mechanisms are still unclear. In this study, we have elucidated that FLs suppress the modulation of NF-κB/MAPK signaling pathways by targeting the inhibition of activating TLR4 in LPS-induced RAW 264.7 cells. Therefore, the transcription and expression of inflammatory cytokines (i.e., TNF-*α*, IL-1*β*, and IL-6) and inflammatory mediator proteins (i.e., iNos and Cox-2) were significantly suppressed by FLs. In addition, an in silico study discovered that eight monomers of FLs showed high-affinity bindings with TLR4. In silico data combined with HPLC results indicated that FLA and FLE, accounting for 44%, were likely the major anti-inflammatory monomers in FLs. In summary, FLA and FLE were proposed as the main anti-inflammatory active cyclopeptides via hindering TLR4/NF-κB/MAPK signaling pathways, suggesting the potential use of food-derived FLs as natural anti-inflammatory supplements in a daily diet.

## 1. Introduction

In recent years, chronic inflammatory diseases such as ulcerative colitis, chronic obstructive pulmonary disease, rheumatoid arthritis, and periodontal disease have increased in prevalence globally. These chronic inflammatory diseases seriously affect the quality of human health and increase the socio-economic burden of people [1,2,3,4]. Studies have revealed that harmful environmental factors, bad lifestyles, and unhealthy diets could contribute to the development of inflammation [5,6,7]. Accumulating evidence has suggested that an imbalanced diet is highly involved in the progression of chronic inflammation. Over-depletion of macronutrients causes sustained activation of inflammatory mediators associated with metabolic diseases, leading to an increase in local and systemic pro-inflammatory biomarkers and ultimately to the development of chronic low-grade inflammation [8]. Adolph et al. suggested that excessive intake of specific macronutrients, such as carbohydrates and lipids, could exacerbate the inflammatory response in the gut by exploiting innate immune sensors and disturbances in gut microbial metabolism [9]. Moreover, animal and human studies have discovered that micronutrient deficiency diets could cause inflammatory cell infiltration and the expression of cell death receptors associated with organ lesions [10,11,12]. As a consequence, adjusting dietary structure with supplements might be an effective way to alleviate chronic inflammation.

Generally, chronic inflammatory conditions are difficult to characterize and further eradicate. Considering this, it is a key issue to develop a natural substance with anti-inflammatory activity that can be ingested over a long period of time without causing side effects in the body. Drugs like nonsteroidal anti-inflammatory drugs (NSAIDs) and glucocorticoids are commonly used to treat inflammatory conditions. However, they have certain side effects, such as resistance and dependence [13]. Screening natural foods for food-borne anti-inflammatory ingredients is a good strategy to reduce inflammation through long-term intake. Several studies have found a strong link between diet and inflammatory prevention and alleviation, such as flavonoids, polyphenols, and unsaturated fatty acids [14]. Rong et al. revealed that the polymethoxyflavonoid nobiletin in the citrus peel could alleviate the inflammatory response by enhancing autophagy through activation of the IL-6/STAT3/FOXO3a pathway in macrophages [15]. Menu et al. discovered that the polyphenol acertannin significantly attenuated lipopolysaccharide (LPS)-induced production of pro-inflammatory cytokines, mediators, and reactive oxygen species by inhibiting NF-κB activation and activating nuclear factor E2-related factor 2 and haem oxygenase 1 [16]. Monmai et al. reported that unsaturated fatty acids in *Asterias amurensis* effectively suppressed LPS-induced inflammatory responses by blocking the NF-κB to MAPK pathway [17]. Therefore, the use of natural functional ingredients in the diet could alleviate chronic inflammation in vivo.

Flaxseed is a special oilseed that is rich in *α*-linolenic acids and a variety of bioactive substances such as lignan, linusorb, polysaccharides, dietary fiber, and protein [18]. Therein, flaxseed linusorbs (FLs) are a type of unique cyclic peptide accounting for around 0.1% and 0.3% of flaxseed and flaxseed oil, respectively [19,20]. In recent years, methods for isolating FLs have become increasingly sophisticated [21,22,23]. Furthermore, natural food-derived FLs have been found to have multiple bioactivities, such as cell cycle arrest, anti-cancer, and anti-bacterial activities in vitro and in vivo [24,25,26]. FLs were reported to have a noteworthy immunosuppressive effect, which could decrease osteoclastogenesis by regulating the expression of *c-fos* [27,28]. In addition, previous studies also mentioned that FLs could inhibit LPS-induced THP-1 macrophages and alleviate gastritis, enteritis, and hepatitis [29,30]. Therefore, FLs have tremendous promise as anti-inflammatory dietary supplements. However, the main anti-inflammatory mechanism of FLs and their main functional monomers have not been elucidated yet.

In this study, the anti-inflammatory mechanism of FLs on LPS-induced RAW 264.7 macrophages was investigated, and the receptor proteins targeted by FLs during anti-inflammatory activity were identified. Moreover, the affinity index of major functional FL monomers binding to the LPS receptor was predicted by molecular docking, which provides a theoretical basis for exploring the anti-inflammatory FL monomers and their mechanisms, as well as evaluating their anti-inflammatory efficacies. The proven in silico results lay the scientific foundation for promoting the application of FLs monomers with considerable anti-inflammatory activity as functional active ingredients in the food, pharmaceutical, and cosmetic industries.

## 2. Materials and Methods

### 2.1. Materials

LPS, dexamethasone (Dex), and dimethyl sulfoxide (DMSO) were purchased from Sigma (St. Louis, MO, USA). FLs were purchased from Prairie Tide Chemicals (Saskatoon, SK, Canada).

### 2.2. Preparation of FLA and FLE

FLA and FLE were separated and identified according to previous methods [23,31]. The crude flaxseed oil was extracted as follows: 500 mL of crude flaxseed oil mixed with 100 mL of silica gel was first loaded onto a glass column and subjected to flash column chromatography with several solvents: 500 mL of 100% hexane; 300 mL of 20% ethyl acetate (EtOAc) in hexane; 300 mL of 50% EtOAc in hexane; 300 mL of 100% EtOAc (A); and 300 mL of 10% methanol in dichloromethane (B). Fractions A and B, concentrated with crude powder, were concentrated in a rotary evaporator at 40 °C, the dry mixture was then dissolved in 100 mL of methanol and centrifuged at 8000 rpm for 15 min at 25 °C afterward. The supernatant was collected, concentrated, and stored for further purification. High-performance liquid chromatography (HPLC) was performed on an Agilent 1260 LC system (Agilent Technologies, Wilmington, DE, USA) with a manual injector with a photo-diode array (PDA) detector set at 214 nm and equipped with a reversed phase Kinetex^®^ Phenyl-hexyl column (250 mm × 21.2 mm, 5 μm, Phenomenex Inc. Torrance, CA, USA). The HPLC elution conditions were as follows: initial acetonitrile (ACN) to water ratio of 40%, final ACN ratio of 80%, elution time of 21 min, flow rate of 16 mL/min, sample loading of 12.5 mg at a concentration of 80 mg/mL (methanol).

### 2.3. Cell Cultures

Raw 264.7 macrophages (CL-0190) were kindly provided by Procell Life Science & Technology (Wuhan, China) and cultured in Dulbecco’s modified eagle medium (DMEM) media supplemented with 10% of fetal bovine serum (FBS) and 1% of penicillin/streptomycin (P/S) at 37 °C in a humidified atmosphere containing 5% of CO_2_. Cells in the exponential growth phase were used for experiments. DMEM, FBS, P/S, and phosphate-buffered saline (PBS) were purchased from Gibco (Carlsbad, CA, USA).

### 2.4. Cell Viability Assays

To evaluate the potential cytotoxic effects of FLs, a cell viability assay was performed by cell counting kit-8 (CCK-8). The cells were seeded in a 96-well plate at 1 × 10^4^/well density and cultured overnight. The cells were incubated with FLs at concentrations (6.25, 12.5, 25, 50, and 100 μg/mL) for 24 h, then 10 μL of CCK-8 solution was added and incubated for another 4 h. The optical density was measured at 450 nm with the culture medium as the blank. The cell viability was calculated according to the following Equation (1),

(1) $$Cell\;viability\;\%=100\times \frac{\left[OD\;of\;the\;experimental\;group-OD\;of\;the\;blank\;group\right]}{\left[OD\;of\;the\;control\;group-OD\;of\;the\;blank\;group\right]}$$

### 2.5. Determination of NO Secretion

The amount of NO released by Raw 264.7 macrophages was determined using a NO assay kit (Beyotime, Shanghai, China) following the manufacturer’s protocol. Firstly, the cells were seeded into a 96-well plate (4 × 10^4^/well) and cultured overnight. LPS (0.2 μg/mL) and a series of concentrations of FLs were cotreated with cells for 24 h. The cell culture medium was then collected, and the NO amount was determined by the Griess assay. Briefly, 50 μL of supernatant from different treatments were collected, and the enzymatic conversion of the supernatant nitrate to nitrite by nitrate reductase was determined by colorimetric assay at 540 nm.

### 2.6. ELISA Assays for Cytokine Determination

The amount of protein expression of tumor necrosis factor-α (TNF-*α*), interleukin-1*β* (IL-1*β*), and interleukin-6 (IL-6) was evaluated by a Sandwich ELISA kit (Elabscience Biotechnology, Wuhan, China) according to the manufacturer’s protocol. Firstly, cells were seeded into a 96-well plate (5 × 10^4^/well) and cultured overnight. LPS (0.2 μg/mL) and a series of concentrations of FLs were cotreated with cells for 24 h without serum. A supernatant of 100 μL from each group was added to each well of the enzyme-labeled adsorption plate and incubated at 37 °C for 90 min. Biotinylated detection antibody (100 μL) was then added into each well after removing the supernatant media (1 h, 37 °C). Subsequently, horseradish peroxidase conjugate working solution (100 μL) was added to each well after washing three times with PBS (20 min, 37 °C). After washing 5 times, substrate reagent (90 μL) was added to each well and incubated for 15 min. Finally, stop solution (50 μL) was added, and the absorption of each well was immediately measured at 450 nm.

### 2.7. Quantitative Real-Time PCR

Raw 264.7 macrophages were seeded into a 6-well plate (1.2 × 10^6^/well) and cultured overnight. LPS (0.2 μg/mL) and a series of concentrations of FLs have treated cells for 24 h. Total mRNA from cells was then extracted using Trizol (Beyotime, Shanghai, China), and cDNA was immediately synthesized using Beyort III CDNA First Chain Combine Kits (Beyotime, Shanghai, China) according to the manufacturer’s instructions. The cDNA was used for quantitative real-time PCR with ChamQ Universal SYBR qPCR Master Mix (Vazyme, Nanjing, China) to detect mRNA levels of IL-1*β*, IL-6, TNF-*α*, iNos, and Cox-2, respectively. Glyceraldehyde-3-phosphate dehydrogenase (GAPDH) was used as an internal control. Primer sequences used in this study are listed in Table 1.

### 2.8. Western Blot Analysis

Raw 264.7 macrophages were seeded into a 6 cm plate (1.5 × 10^6^/well) and cultured overnight. LPS (0.2 μg/mL) and a series of concentrations of FLs were cotreated with cells for 24 h. Then, cells were lysed with radio-immunoprecipitation assay (RIPA) lysis buffer containing 1% of protease and phosphatase inhibitors (Beyotime, Shanghai, China). The total protein concentrations were determined using a bicinchoninic acid (BCA) protein assay kit (Beyotime, Shanghai, China), and equal amounts of protein were loaded on 10% of SDS-polyacrylamide gel electrophoresis (Beyotime, Shanghai, China). Subsequently, proteins were transferred to PVDF membranes (Merck Millipore, Darmstadt, Germany) and then blocked in the skim milk for 1 h at atmospheric temperature. The primary antibodies against β-actin, iNos, Cox-2, IκBα, p-P65, p-P38, p-ERK, and TLR4 (Cell Signaling, Boston, MA, USA) were incubated in the membrane overnight at 4 °C, respectively. The immunoreactive bands are visualized on a gel imaging system (Tanon Science & Technology, Shanghai, China) after incubation of the HRP-secondary antibody.

### 2.9. Confocal Assays

Raw 264.7 macrophages (5 × 10^4^/well) were seeded in a 3.5 cm glass bottom plate and treated with LPS + FLs for 12 h. Subsequently, cells were fixed with paraformaldehyde solution (4%) at atmospheric temperature for 30 min and then blocked with immunostaining blocking buffer (Beyotime, Shanghai, China) for 45 min. Cells were incubated with the monoclonal antibody p-P65 (1:100, Cell Signaling Technology, Boston, MA, USA) overnight at 4 °C. FITC-goat anti-rabbit IgG (1:1000, Beyotime, Shanghai, China) stained cells for 1 h, avoiding light. 4′,6-Diamidino-2-phenylindole (DAPI) (Beyotime, Shanghai, China) was added to each plate and incubated for 5 min. Finally, cells were observed under a confocal laser scanning microscope (CLSM, LSM880 Airyscan, Zeiss, Jena, Germany) with a 10× objective lens with NA 0.45 and a 63× oil objective lens with NA 1.4.

### 2.10. Molecular Docking of FLs with TLR4

To further investigate the anti-inflammatory role of monomer in the FLs, the structure of the FL monomer was plotted in ChemDraw 20.0, and the 3D structure of TLR4 (code: 2z64) was downloaded from the protein data bank (PDB) database (http://www.rcsb.org/pdb/, accessed on 29 September 2022). Molecular docking simulations between FLs and TLR4 were carried out in the Sybyl-X Molecular Modeling software packages (Version 2.0, TRIPOS Associates, St. Louis, MO, USA), which allowed the movement of hydrogen and heavy atoms within the protein, as well as the removal of water molecules and the addition of hydrogen atoms, with other parameters set to default values. In the docking results, only the active cavity and pocket of TLR4 docking with FLs were shown. The magnitude of the interaction force between FLs and TLR4 was assessed based on the total score, crash value, polar value, and the number of H-bonds.

### 2.11. Statistical Analysis

All experiments were performed in triplicate, and data are presented as means ± standard deviation (SD). Data analyses were performed using the SPSS 27.0 software (SPSS Inc., Chicago, IL, USA). The significant differences among groups were evaluated by one-way ANOVA analysis, where a significant difference (*p* < 0.05) was considered at the 95% confidence level.

## 3. Results

### 3.1. Effect of FLs on Cell Viability and Inhibition of NO

**Figure 1:**
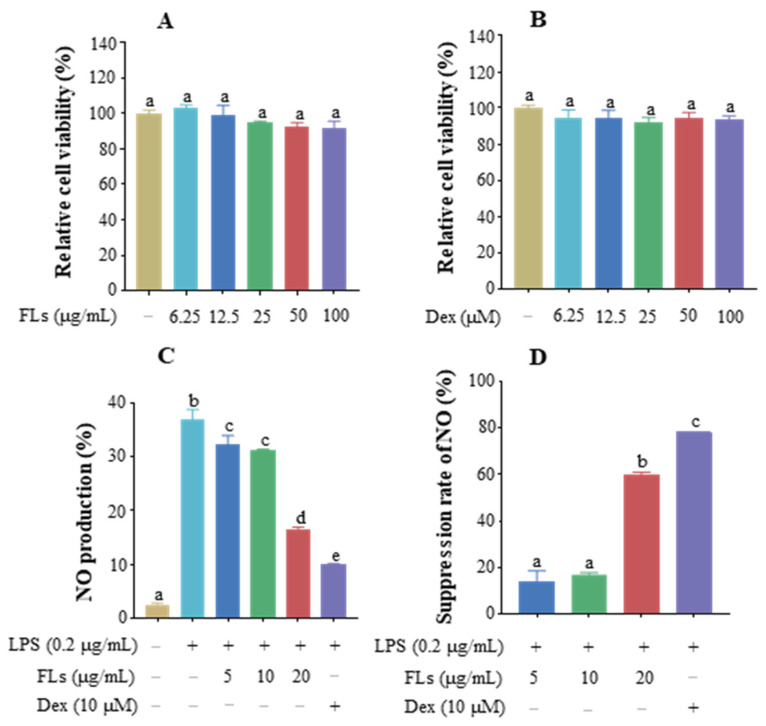
Effect of FLs (**A**) and Dex (**B**) on Raw 264.7 macrophage viability, the effect of FLs on NO production (**C**), and the inhibitory rate of FLs in NO release (**D**) in LPS-induced Raw 264.7 macrophages. Different lowercase letters indicate that the data are statistically different (*p* < 0.05).

As Figure 1A illustrated, the cell viability of FLs in Raw 264.7 macrophages ranged from 91.5% to 103.4% at concentrations of 0–100 μg/mL with no significant difference. This result indicated that there was no cytotoxicity of FLs in Raw 264.7 macrophages below 100 μg/mL. Subsequently, various concentrations of FLs (5, 10, and 20 μg/mL) were selected for the following experiments with dexamethasone (Dex) as the positive control. The CCK8 results showed that cell survival ranged from 92.0% to 100% at concentrations of 0–100 μM with no significant differences (Figure 1B).

Excessive production of NO is an important indicator of an organism’s inflammatory response, which can lead to vasodilation and impaired microcirculation, promoting inflammation [32]. The results showed that FLs significantly inhibited NO production in a dose-dependent manner (Figure 1C). The secretion of NO decreased by 13.8% and 16.5% at 5 and 10 μg/mL, respectively. Suppression of NO increased by 60.1% with 20 μg/mL of FLs treatment, which effect was close to the 78.0% restraint of NO by Dex (Figure 1D). In summary, FLs showed no cytotoxicity until 100 μg/mL and prohibited NO production in a dose-dependent manner.

### 3.2. Effects of FLs on the Inhibiting Secretion of Pro-Inflammatory Mediators and Cytokine

**Figure 2:**
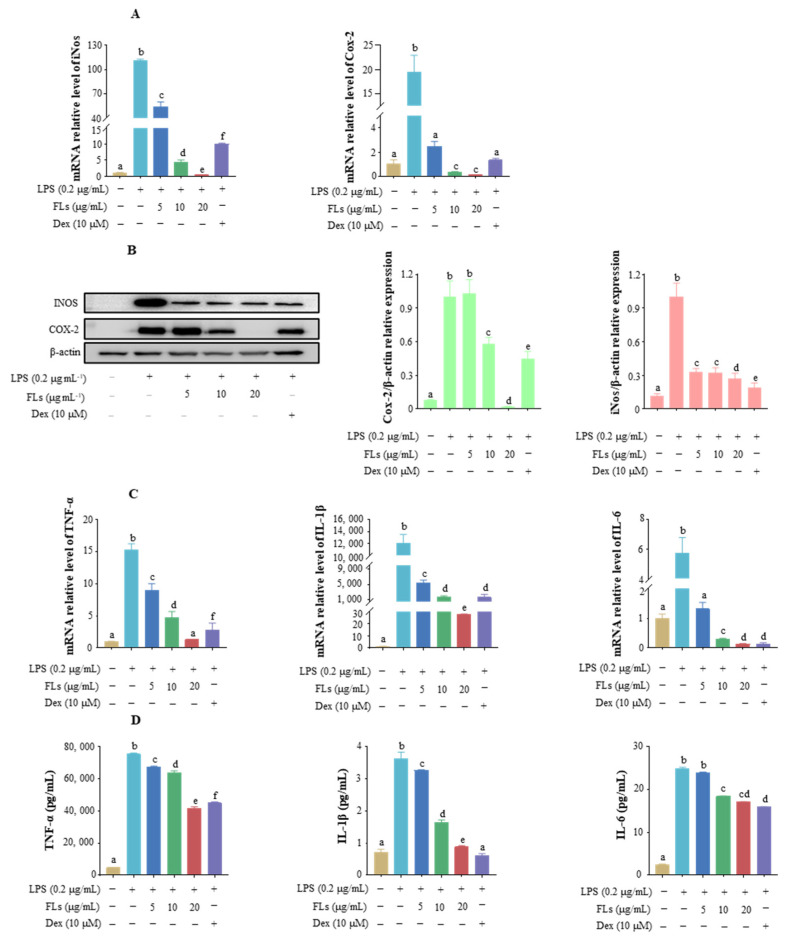
Effect of FLs on the production of pro-inflammatory mediators iNos and Cox-2 expression mRNA level (**A**), protein level (**B**), and pro-inflammatory factors TNF-*α*, IL-1*β*, and IL-6 mRNA level (**C**), protein level (**D**) in the LPS-induced Raw 264.7 macrophages. Different lowercase letters indicate that the data are statistically different (*p* < 0.05).

iNos is a necessary enzyme for NO production, which has toxic effects on host tissues [33]. The Cox-2 enzyme is required to produce prostaglandin E2 (PGE-2), which can cause severe pain in humans during inflammation [34]. Therefore, inhibition of iNos and Cox-2 is the key to alleviating inflammatory symptoms. Compared to the control group, the mRNA levels of iNos and Cox-2 in the LPS group were enhanced 110.5-fold and 19.5-fold, respectively (Figure 2A). Meanwhile, the relative transcription level of iNos was reduced by 51.1%, 96.1%, and 99.6% in LPS-induced Raw 264.7 macrophages with 5, 10, and 20 μg/mL of FL intervention, respectively. The mRNA level of Cox-2 was reduced with increased concentrations of FLs by 87.3%, 98.3%, and 99.4%, respectively. The protein levels of iNos and Cox-2 were suppressed by FLs in LPS-induced cells, which is in line with the mRNA level trend. The expression of iNos and Cox-2 was decreased by 73.4% and 98.1% with 20 μg/mL of FL intervention, respectively (Figure 2B). A better inhibition of Cox-2 was observed in FLs (20 μg/mL, 98.1%) than that of the Dex group (10 µM, 54.9%). Given these data, FLs showed a significant effect on the suppression of pro-inflammatory mediator markers, which might help to ameliorate inflammation.

Pro-inflammatory cytokines (e.g., TNF-*α*, IL-6, and IL-1*β*) released by cells against outward stimulation are a group of small-molecule proteins that can promote inflammatory responses [35]. Compared to the control group, the transcript levels of TNF-*α*, IL-1*β*, and IL-6 in the LPS group increased by 15.3-fold, 11997.7-fold, and 5.7-fold, respectively (Figure 2C). Compared to the LPS group, the mRNA levels of TNF-*α*, IL-1*β*, and IL-6 reduced by 91.4%, 99.8%, and 97.7% with high-concentration treatment of FLs, respectively. (Figure 2D). Furthermore, the secretion of TNF-*α*, IL-1*β*, and IL-6 in the low, medium, and high concentration groups was also reduced by 10.3%, 15.6%, and 44.6%; 9.9%, 54.7%, and 75.5%; and 4.2%, 26.0%, and 31.2%, respectively. The inhibitory effect of FLs on TNF-*α* expression at high concentration (20 μg/mL) was better than that of Dex (40.3%), while the repression of IL-1*β* and IL-6 protein expression was slightly lower than that of Dex (83.1% and 36.0%, respectively). Taken together, FLs showed a desirable effect on inhibiting pro-inflammatory cytokines under 20 μg/mL.

### 3.3. Effects of FLs on Blocking NF-κB/MAPK Signaling Pathway

NF-κB is a dimeric nuclear transcription factor consisting of P50 and P65 subunits that can activate the transcription of inflammatory factors and mediators, such as TNF-*α*, IL-1*β*, IL-6, iNos, and Cox-2 [36]. Western blot results showed that FLs suppressed the expression of p-NF-κB in a dose-dependent manner while the NF-κB inhibitor IκB*α* in LPS-induced cells was increased. The levels of p-NF-κB protein in LPS-induced cells fell by 13.5%, 48.0%, and 52.0% when treated with 5, 10, and 20 g/mL of FLs, respectively. However, the level of IκB protein increased by 6.4% and 104.5% when treated with 10 and 20 g/mL of FLs, respectively. Moreover, FLs suppressed p-NF-κB better than positive control medicines Dex (43.5%) at high doses.

**Figure 3:**
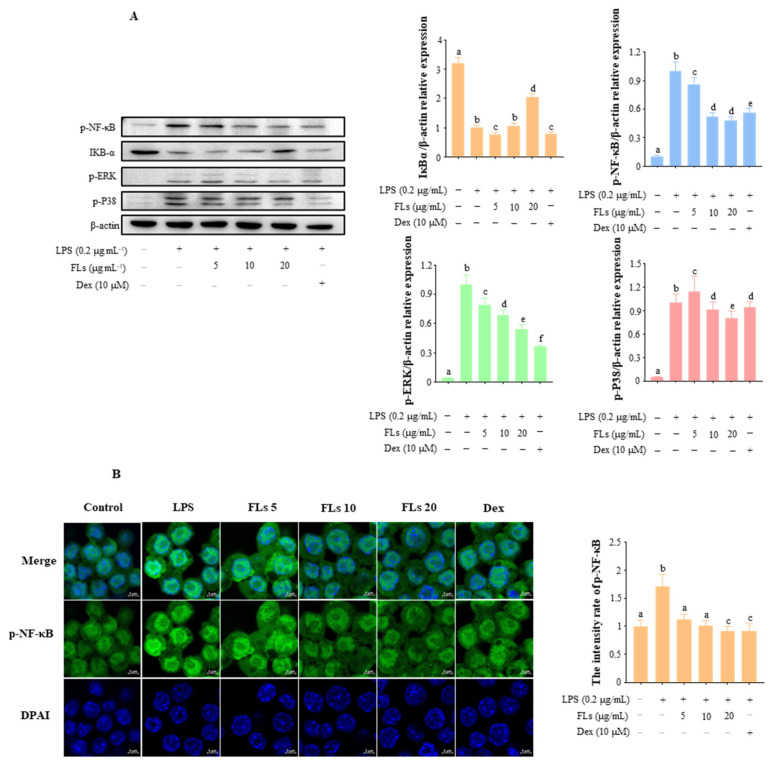
Effect of FLs on the LPS-induced NF-κb/MAPK signaling pathway in Raw 264.7 macrophages (**A**). Effect of FOs on LPS-induced nuclear translocation of p-NF-κB in Raw 264.7 macrophages, scale bar: 5 μm (**B**). Different lowercase letters indicate that the data are statistically different (*p* < 0.05).

Blocking the activation of extracellular signal-regulated kinase (ERK) and P38 in the MAPK signaling pathway is another effective means to suppress inflammation [37,38]. Compared to the control group, the protein levels of p-P38 and p-ERK in the LPS group were enhanced 22.9-fold and 18.3-fold, respectively (Figure 3A). Moreover, the level of p-P38 protein in LPS-induced Raw 264.7 macrophages treated with low, medium, and high concentrations of FLs decreased by 20.6%, 31.6%, and 45.6%, respectively. Meanwhile, the level of p-ERK protein in the medium and high concentration groups decreased by 8.8% and 19.3%, respectively. The high dosage of FLs suppressed p-ERK protein production more than Dex (5.8%) but had a significantly lower inhibitory impact on p-P38 (63.5%). Therefore, FLs could prevent the inflammatory response by inhibiting the NF-κB/MAPK signaling pathway.

CLSM results showed that the fluorescence intensity of p-NF-κB significantly increased in the nuclear fraction (Figure 3B). FLs reversed the activation of p-NF-κB in a dose-dependent manner. The suppression of p-NF-κB fluorescence intensity by high concentrations of FLs was superior to that of Dex (79.7%). Thus, FLs could inhibit the inflammatory response by restricting NF-κB phosphorylation and p-NF-κB nuclear translocation in a dose-dependent manner.

### 3.4. FLs Target TLR4 Protein Directly

**Figure 4:**
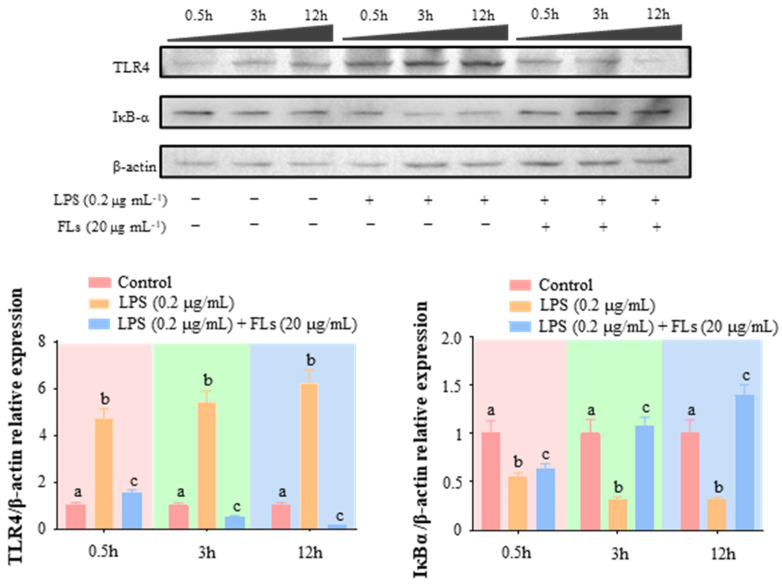
Effect of FLs on LPS-induced inflammatory signaling pathway-related proteins in Raw 264.7 macrophages at different time scales. The bar graph represents the relative expression of the target protein over the *β*-actin protein, where the relative expression of the protein in the control group was set to 1. Different lowercase letters indicate that the data are statistically different (*p* < 0.05).

Foreign inflammation-inducing substances such as LPS can initiate the downstream NF-κB/MAPK inflammatory cascade by binding to the transmembrane protein TLR4 on the cell surface, which promotes gene transcription and protein expression of inflammation-related factors, resulting in the development of the body’s inflammatory response [39]. The time-course data indicated that FLs blocked the activation of TLR4 from 0.5 h (Figure 4). LPS continuously triggered the expression of TLR4 from 0.5 h to 12 h. However, FLs suppressed TLR4 expression by 67.2%, 90.4%, and 97.7% at 0.5 h, 3 h, and 12 h, respectively. The elevating rate of IκB expression was 15.1%, 237.0%, and 343.5% at 0.5 h, 3 h, and 12 h, respectively. Hence, FLs directly suppressed the activation of TLR4, resulting in inhibiting NF-κB/MAPK signaling.

### 3.5. In Silico Affinity of FLs Binding to TLR4

Molecular docking is primarily a theoretical simulation method for studying intermolecular interactions and predicting their binding patterns and affinities [40]. The interaction force of twelve isolated FLs monomers with TLR4 was predicted in silico, and the structural features of FLs monomers are shown in Table S1. Four key indicators reflected the affinity directly between FLs and the target protein, including the total score, crash value, polar value of the binding complex, and the number of H-bonds. The total score indicates the binding affinity between the ligand and the receptor. The crash value is the crash parameter of the docking process. The polar value is the polarity parameter of the docking. The number of H-bonds indicates the binding strength of the ligand to the protein. Eight kinds of FLs had a high total score (>7) and 2–5 hydrogen bonds, indicating a high affinity for TLR4 (Table 2). These eight FLs were named flaxseed linusorb H, L, N, G, F, B, M, and E, respectively. The crash value ranging from −7.96 to −1.88 and the polar value ranging from 1.08 to 4.37 indicated good adaptability of pocket docking between these eight FLs and TLR4.

**Figure 5:**
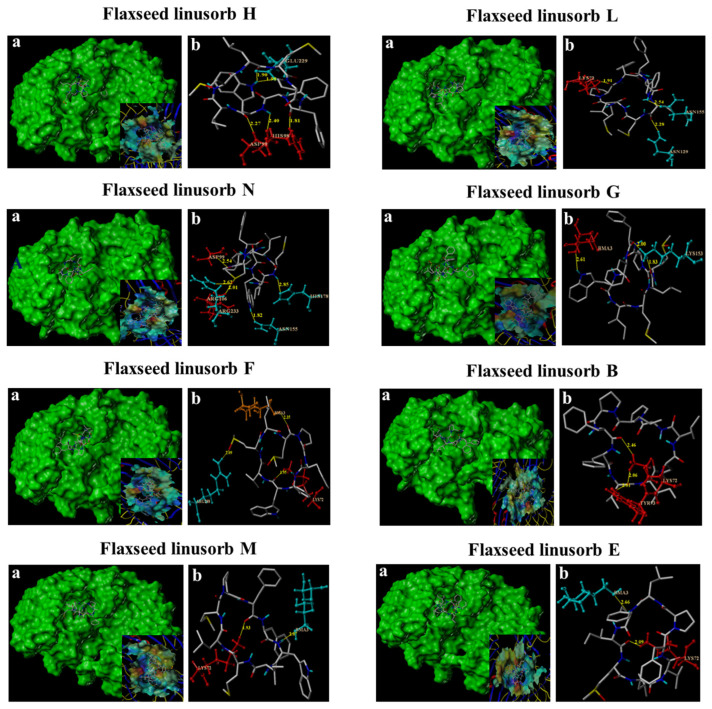
The cavity formed locally by TLR4 with each flaxseed linusorb monomer and the virtual pocket formed by force (**a**) and amino acid number, number of hydrogen bonds, and distance between TLR4 and flaxseed linusorbs forming a hydrogen bond (**b**).

As Figure 5 illustrates, the main residues of FLH binding to TLR4 are HIS98, ASP99, and GLU299, and the distances (R) from FLH to TLR4 were 2.40/1.81 Å, 2.27 Å, and 1.90/1.98 Å, respectively. The main residues of FLL binding to TLR4 are LYS72, ASN129, and ASN155, and the distances (R) from FLL to TLR4 were 1.91 Å, 2.28 Å, and 2.54 Å, respectively. The main residues of FLN binding to TLR4 are ASP99, ARG106, ASN155, HIS178, and ARG233, and the distances (R) from FLN to TLR4 are 2.54 Å, 2.01 Å, 1.82 Å, 2.85 Å, and 2.62 Å, respectively. The major residues binding to TLR4 by FLG are BMA3 and LYS153, and the distances (R) from FLG to TLR4 are 2.61 Å and 1.83/2.00 Å, respectively. The major residues binding to TLR4 are BMA3, LYS72, and ARG288, and the distances (R) from FLF to TLR4 are 2.35 Å, 1.95 Å, and 2.09 Å, respectively. FLB binding to TLR4 with the major residues LYS72 and TRY7 has distances (R) of 2.06/2.64 Å and 2.01 Å from FLB to TLR4, respectively. FLM binds to TLR4 with the major residues BMA3 and LYS72, and the distances (R) from FLM to TLR4 are 2.07 Å, 2.27 Å, and 1.93 Å, respectively. FLE binds to TLR4 with the major residues BMA3 and LYS72, and the distances (R) from FLE to TLR4 are 2.66 Å and 2.09 Å, respectively.

### 3.6. Anti-Inflammatory Activity of FLA and FLE

**Figure 6:**
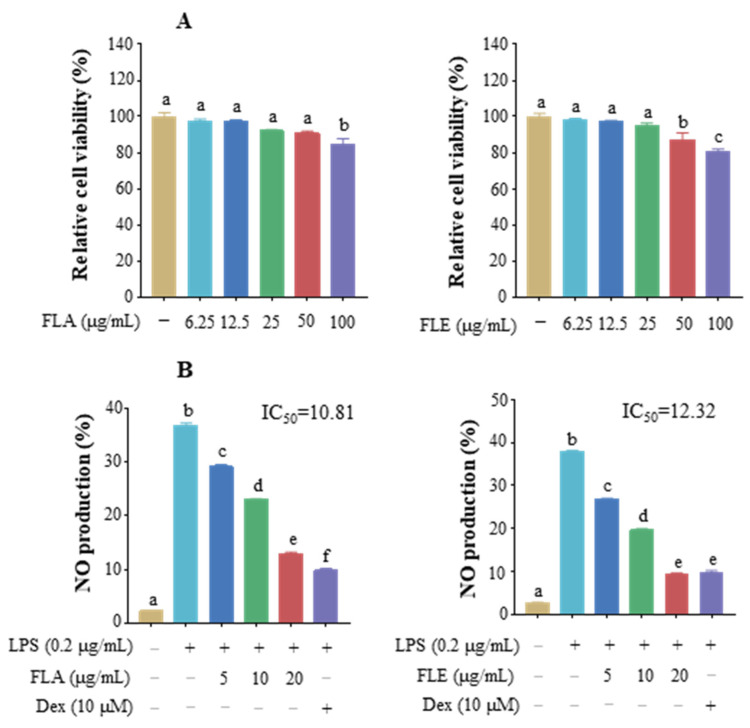
Effects of FLA and FLE on Raw 264.7 macrophage viability (**A**) and NO production (**B**) in the LPS-induced Raw 264.7 macrophage inflammation model. Different lowercase letters indicate that the data are statistically different (*p* < 0.05).

Flaxseed linusorb monomers were further analyzed by HPLC, and their proportions are shown in Table 2. The anti-inflammatory activity of two major monomers, FLA (23.6%) and FLE (21.1%), with the highest proportion in the mixed FLs, was further investigated. The total score of FLE and FLA binding to the TLR4 protein was 7.13 and 5.07, respectively. CCK8 results showed that the cell survival rate with FLA and FLE treatment ranged from 84.4% to 100% and 80.7% to 100% at concentrations of 0–100 μg/mL (Figure 6A). NO was used as an indicator to investigate the anti-inflammatory activity of FLA and FLE, which showed that both FLA and FLE prohibited NO production in a dose-dependent manner at concentrations of 5, 10, and 20 μg/mL (Figure 6B). FLA and FLE at higher concentrations inhibited NO by 51.8% and 74.7%, respectively. The IC_50_ of NO production was 12.32 and 10.81 μM for FLA and FLE treatment, respectively. This trend is in line with the in silico data showing that FLE has a higher affinity for TLR4 than FLA, resulting in a higher inhibition of NO production. These results further demonstrated that FLA and FLE were the main monomers against inflammation.

## 4. Discussion

Inflammation plays a pivotal role in maintaining health as a self-protective immune response. However, long-term chronic inflammation leads to immune dysregulation and the accumulation of inflammatory cells in tissues [41]. Furthermore, FLs were found to be the cause of the bitter taste of flaxseed oil, which could be removed to obtain “sweet” flaxseed oil. Hence, studying the anti-inflammatory activity of FLs not only has excellent value in improving the application of these by-products from the flaxseed oil refining process, but also provides a new strategy for dietary intervention in preventing inflammatory diseases.

TLR4 is a transmembrane protein receptor that binds with LPS or other substances that trigger inflammation [42]. The activation of TLR4 stimulates downstream inflammatory cascades such as NF-κB and MAPK cell signaling. Canonical NF-κB activation involves the phosphorylation of inhibitory molecules, IκB*α* kinase, and nuclear translocation of five different NF-κB DNA-binding subunits (i.e., p65, REL, RELB, p50, p52) to form various DNA-binding dimers [43,44]. These DNA-binding dimers reprogram gene expression and activate pro-inflammatory mediators such as TNF-*α*, IL-1*β*, IL-6, Cox-2, and iNos [45,46]. Therefore, inhibition of the NF-κB signaling pathway plays a crucial role in regulating inflammation and immunity. You et al. found that pear pomace ethanol extracts alleviated NC/Nga mice to induce atopic dermatitis-like skin lesions by regulating ERK1/2 and NF-κB phosphorylation and reducing iNos and Cox-2 overexpression in RAW 264.7 cells [47]. Jeong et al. demonstrated that *Aster glehni* extract improved the symptoms of potassium oxonate-induced hyperuricemia in rats by inhibiting the TLR4/MyD88/NF-κB signaling pathway and modulating renal transporter proteins [48]. MAPK included extracellular signal-regulated kinase (ERK) and two stress-activated protein kinase (SAPK) families, c-Jun N-terminal kinase (JNK) and p38. MAPK phosphorylates threonine/tyrosine residues translocated into the nucleus and bound transcription factors such as c-Jun and c-Fos, triggering the transcription of pro-inflammatory and growth differentiation factors, thereby responding to inflammatory stimuli by regulating cell differentiation and apoptosis [49,50]. Effective suppression of MAPK signaling pathways was also essential for the alleviation of inflammation. Kopalli et al. found that cordycepin isolated from the potential medicinal fungi *Cordyceps militari* could overregulate ERK 1/2, P38, and JNK to restore alterations in serum biochemical parameters in aged rats and ameliorate aging-related of testicular inflammation [51]. Ye et al. elucidated that a modifier of the dietary flavonoid Luteolin (LUA) exhibited superior anti-inflammatory activity against LPS-induced RAW 264.7 cells by inhibiting the expression of JNK and P38 [52], thereby reducing the production of inflammatory factors and reactive oxygen species. This study suggested that FLs restricted NF-κB and MAPK signaling pathways to ameliorate the LPS-induced inflammatory response in Raw 264.7 macrophages. Similarly, Zou et al. concluded that FLA and FLB could prevent LPS-induced inflammation in THP-1 cells by down-regulating key proteins of the NF-κB signaling pathway (p-IKK*α*/*β*, p-IκB*α*, p-NF-κB) [29]. Ratan et al. reported that FLs could suppress the transcription of proinflammatory mediators, such as iNos, Cox-2, and TNF-*α,* by blocking the NF-κB signaling pathway [30]. Interestingly, Chen et al. discovered that the cyclopeptide Heterophyllin B from *Pseudostellaria heterophylla* might ameliorate ulcerative colitis in DDS-induced mice by lowering inflammatory factor production and macrophage infiltration in the colon [53]. Ni et al. discovered that hydrolyzed chicken meat extract containing cyclopeptide improved LPS-induced inflammation and oxidative stress in macrophages by modulating MAPK and NF-κB pathways as well as STAT6 and AKT pathways [54]. In this study, FLs were first found to alleviate the inflammatory response by targeting the suppression of TLR4 activation in the TLR4/NF-κB/MAPK signaling pathway.

Molecular docking is the most popular computational structure-based drug design method and has become an effective computer-aided technique for drug discovery [55,56,57]. The spatial and energetic match allows molecular docking to predict the conformation of small molecule ligands in the appropriate target binding site with high accuracy. Results indicated that FLs have the potential to inhibit TLR4 activation by competitively binding TLR4 to LPS. Previous studies showed that the binding of LPS to TLR4 activated inflammatory pathways, which, in turn, promoted the expression of associated inflammatory factors [58,59]. The expression of inflammatory factors further promoted the expression and activation of TLR4. When the binding of LPS to TLR4 was competitively inhibited by FLs, both activity and expression of TLR4 were reduced, which is consistent with the inhibition of TLR4 expression by FLs in the Western blot results. In addition, eight kinds of FL monomers showed high affinities to bind with TLR4. However, FLH, FLL, and FLN monomers with high total scores accounted for a small proportion of FLs. FLA and FLE, accounting for over 40% of FLs in total, showed a good binding index score (5.07 and 7.13). Concerning the anti-inflammatory effect of FLA and FLE on inhibiting NO, FLE (IC_50_ = 10.81 μM) was superior to FLA (IC_50_ = 12.32 μM), which is consistent with the molecular docking results. Although FLA and FLE showed good activity in inhibiting NO as the signature product of inflammation, proving whether the same anti-inflammatory effect could be achieved at the same effective dose for our bodies in real life still needs to be further verified by animal studies. Interestingly, Zou et al. found that the anti-inflammatory effect of FLA was superior to that of FLB, and the suppression of NO by FLA at 2.08 μg/mL was 40%, which is comparable to that of FLB at 4.23 μg/mL [29]. Similarly, FLA (IC_50_ = 12.5 μg/mL) inhibited NO better than FLB (IC_50_ = 37.3 μg/mL). Although the molecular docking score index of FLB-TLR4 was higher than that of FLA, FLB was not the major component of FLs in this study. It is worth noting that FLs have shown their anti-inflammatory effects, which could suppress NO at 100 μg/mL and 200 μg/mL by around 30% and 50%, respectively [30]. In this study, the suppression of NO by 20 μg/mL of FLs reached approximately 60%. This might be due to the various compositions and proportions of the blended FLs. In addition, the proportion of FLs with a total score of 7 for single monomers reached 54.4% according to molecular docking results, which led to a perfect anti-inflammatory effect of FLs.

## 5. Conclusions

The targeted inhibitory effect of FLs on the inflammatory response of LPS-induced Raw 264.7 macrophages was investigated, where FLs could inhibit NF-κB/MAPK signaling pathway transduction and downregulate the secretion of pro-inflammatory factors and the expression of pro-inflammatory mediators. FLs could also exert impressive anti-inflammatory activity by targeting TLR4 expression, which showed a promising prospect for developing natural anti-inflammatory agents. Moreover, the in silico binding effect of FL monomers on the inflammatory target protein TLR4 further demonstrated that FLA and FLE were proposed as major anti-inflammatory monomers according to HPLC results. However, it is expected to explore the structure-anti-inflammation relationship for the monomer of FLs in future studies, involving the development of efficient isolation methods for different monomeric compounds of FLs from flaxseed oils. Furthermore, the in vivo anti-inflammatory effect of FLs still needs to be further evaluated before commercialization.

## Figures and Tables

**Table 1:** Primer sequences used for polymerase chain reaction (PCR).

Target		Sequence (5′ to 3′)
iNos	Forward	GGAAGAGACACTACTGCTGGT
	Reverse	GAACTGGAGGTACTGCTGGAGC
Cox-2	Forward	TTTCTACCAGAAGGGCAGGAT
	Reverse	TATCACAGGCTTCCATTGACC
IL-1*β*	Forward	TGCCACCTTTTGACAGTGATG
	Reverse	AAGGTCCACGGGAAAGACAC
IL-6	Forward	CCCCAATTTCCAATGCTCTCC
	Reverse	CGCACTAGGTTTGCCGAGTA
TNF-*α*	Forward	ATGGCCTCCCTCTCATCAGT
	Reverse	TTTGCTACGACGTGGGCTAC
GAPDH	Forward	CCAGCTACTCGCGGCTTTA
	Reverse	GTTCACACCGACCTTCACCA

**Table 2:** Molecular docking score for TLR4-docking flaxseed linusorb monomers and their proportions in FLs analyzed by HPLC.

Compound	Total Score	Crash	Polar	Number of H-Bond	Proportion (%)
FLH	11.97	−7.43	4.37	5	3.84
FLL	11.46	−6.52	2.10	3	5.95
FLN	8.18	−7.96	2.44	5	2.54
FLG	8.07	−2.45	2.08	3	7.44
FLF	8.01	−4.51	2.22	3	4.28
FLB	7.94	−7.17	2.34	3	2.91
FLM	7.16	−3.78	2.05	2	6.39
FLE	7.13	−1.87	1.08	2	21.08
FLC	5.55	−6.69	1.37	3	11.78
FLA	5.07	−4.77	2.53	4	23.56
FLP	4.92	−3.68	2.84	4	6.82
FLO	4.57	−5.42	1.79	3	3.41

## Data Availability

The authors confirm that the data supporting the findings of this study are available within the article.

## Supplementary Materials

The following supporting information can be downloaded at: https://www.mdpi.com/article/10.3390/foods12122398/s1, Table S1: Structural features of flaxseed linusorb monomers.
