# Supplementary material for: The Anti-Inflammatory Mechanism of Flaxseed Linusorbs on Lipopolysaccharide-Induced RAW 264.7 Macrophages by Modulating TLR4/NF-κB/MAPK Pathway
Source: Foods. 2023 Jun 16;12(12):2398. doi: 10.3390/foods12122398 (PMC10297071; doi:10.3390/foods12122398)
Supplement: Supplementary file 1 [file foods-12-02398-s001.zip › foods-2393157-supplementary.pdf]

Supplementary Materials

Table S1. Structural features of FLs monomers.

	FLA	FLE	FLC	FLG	FLP	FLM
Structural formula	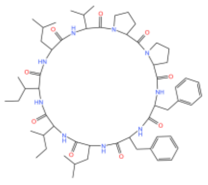	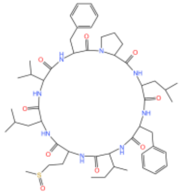	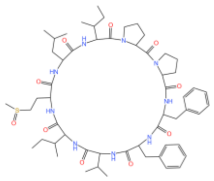	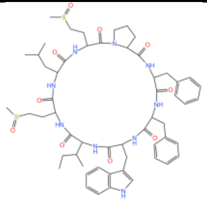	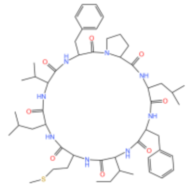	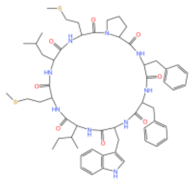
Molecular formula	$C_{57}H_{85}N_9O_9$	$C_{51}H_{76}N_8O_9S$	$C_{56}H_{83}N_9O_{10}S$	$C_{56}H_{75}N_9O_{10}S_2$	$C_{51}H_{76}N_8O_8S$	$C_{56}H_{75}N_9O_8S_2$
Molecular weight	1040.3	977.3	1074.4	1098.4	961.3	1066.4
Amino acid composition	Ile-Leu-Val-Pro-Pro-Phe-Phe-Leu-Ile	MetO-Leu-Val-Phe-Pro-Leu-Phe-Ile	MetO-Leu-Ile-Pro-Pro-Phe-Phe-Val-Ile	MetO-Leu-MetO-Pro-Phe-Phe-Trp-Ile	Met-Leu-Val-Phe-Pro-Leu-Phe-Ile	Met-Leu-Met-Pro-Phe-Phe-Trp-Ile
	FLL	FLF	FLH	FLO	FLB	FLN
Structural formula	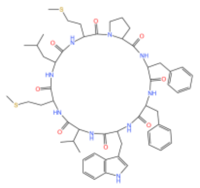	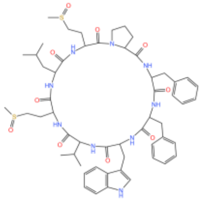	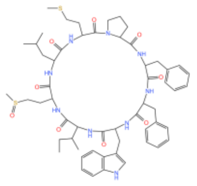	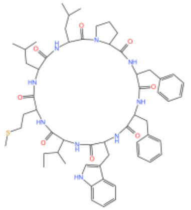	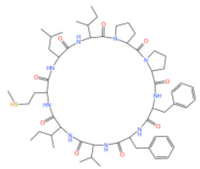	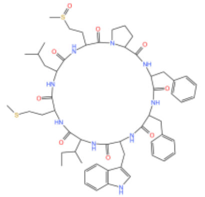
Molecular formula	$C_{55}H_{73}N_9O_8S_2$	$C_{55}H_{73}N_9O_{10}S_2$	$C_{56}H_{75}N_9O_9S_2$	$C_{57}H_{77}N_9O_8S$	$C_{56}H_{83}N_9O_9S$	$C_{56}H_{75}N_9O_9S_2$
Molecular weight	1051.5	1084.4	1082.4	1048.3	1058.4	1082.4
Amino acid composition	Met-Leu-Met-Pro-Phe-Phe-Trp-Val	MetO-Leu-MetO-Pro-Phe-Phe-Trp-Val	MetO-Leu-Met-Pro-Phe-Phe-Trp-Ile	Met-Leu-Leu-Pro-Phe-Phe-Trp-Ile	Met-Leu-Ile-Pro-Pro-Phe-Phe-Val-Ile	Met-Leu-MetO-Pro-Phe-Phe-Trp-Ile
